# Impact of a Virtual, Arts-based Program on Students’ Attitudes Toward Older Adults

**DOI:** 10.1093/geroni/igaf122.1922

**Published:** 2025-12-31

**Authors:** Jenny Kwon, Meghan Young, Yue Li, Amy Elliot

**Affiliations:** University of California, San Francisco, San Francisco, California, United States; Miami University, Oxford, Ohio, United States; Miami University, Oxford, Ohio, United States; Miami University, Oxford, Ohio, United States

## Abstract

As a response to COVID-19, a gerontology service-learning course transitioned from an in-person format to a virtual one, ensuring intergenerational connections could persist despite physical distancing. The course adapted Opening Minds through Art (OMA), an intergenerational art-based program designed to foster relationships between college students and individuals living with dementia while reducing ageism. Through the virtual OMA course, college students and older adults with and without dementia engaged in artistic activities via Zoom. The purpose of this study was to assess changes in students’ perceptions of individuals living with dementia before and after participating in a virtual OMA course. The Allophilia scale measured affection, social comfort and kinship, engagement and enthusiasm toward individuals living with dementia. Self-reported survey data were collected from 130 students enrolled in the course between Fall 2020 and Spring 2022. Pre- and post-assessments were conducted at the start and end of each semester. Results from the paired-sample t-tests show significant increases in overall Allophilia scores (p < .001) and three subscale scores (p < .001) after students’ interaction with individuals living with dementia. Findings from multiple linear regression indicate that pre-scores and prior experience with individuals living with dementia were significant predictors of higher post-Allophilia scores across all domains (p < .001). The effect of pre-score on Allophilia scores was stronger among students without prior experience with individuals living with dementia (p < .05). These findings highlight the effectiveness of virtual intergenerational courses in reducing ageism and shaping positive perceptions of older adults living with dementia.

